# Functional characterization of a short peptidoglycan recognition protein from Chinese giant salamander (*Andrias davidianus)*

**DOI:** 10.18632/oncotarget.21470

**Published:** 2017-10-03

**Authors:** Zhitao Qi, Shisi Ren, Qihuan Zhang, Jun Zou, Qiaoqing Xu, Zisheng Wang, Guo Qiao, Pin Nie, Mingxian Chang

**Affiliations:** ^1^ Jiangsu Key Laboratory of Biochemistry and Biotechnology of Marine Wetland, Yancheng Institute of Technology, Yancheng, Jiangsu, China; ^2^ State Key Laboratory of Freshwater Ecology and Biotechnology, Institute of Hydrobiology, Chinese Academy of Sciences, Wuhan, Hubei; ^3^ Scottish Fish Immunology Research Centre, University of Aberdeen, Aberdeen, UK; ^4^ College of Animal Sciences, Yangtze University, Jingzhou, Hubei, China; ^5^ Key Laboratory of Aquaculture Disease Control, Ministry of Agriculture, Wuhan, Hubei Province, China

**Keywords:** peptidoglycan recognition protein, andrias davidianus, gene clone, functional analysis, Immunology and Microbiology Section, Immune response, Immunity

## Abstract

Peptidoglycan (PGN) recognition proteins (PGRPs) are important pattern recognition receptors (PRRs) involved in immune defense against bacterial infections. In this study, a short PGRP (termed AdPGRP-S1) was cloned and functionally characterized from Chinese giant salamander (*Andrias davidianus*), the largest extant urodela amphibian species. AdPGRP-S1 was 184 aa in length and shared 38.7%-54.9% sequence identities with other vertebrates’ short PGRPs. It contained one typical PGRP domain at the C-terminal region and several conserved amino acid (aa) residues involved in amidase and PGN binding. AdPGRP-S1 was constitutively expressed in all tissues examined, with the highest expression level seen in spleen and intestine. It has been shown that AdPGRP-S1 could bind and degrade Lys-PGN and Dap-PGN. Further, AdPGRP-S1 had antibacterial activity against the Gram-negative bacteria, *Edwardsiella tarda*, and was able to trigger the activation of NF-κB signaling. These results demonstrated that AdPGRP-S1 possesses multiple functions in pathogen recognition, mediating ceullular signaling, and initiating antibacterial response. This is the first functional study of a salamander PGRP, providing insight to further understand the functional evolution of verterbates’ PGRPs.

## INTRODUCTION

Innate immunity is the first line of defense against invading microorganisms, and is triggered by recognition of pathogen associated molecular patterns (PAMPs) through host pattern recognition receptors (PRRs). PAMPs are conserved molecular structures of microorganisms, including bacterial lipopolysaccharide (LPS) and peptidoglycan (PGN), fungal β-1,3-glucan and viral double-stranded RNA (dsRNA) [1]. Several families of PRRs have been identified in vertebrates, including toll-like receptors (TLRs), nucleotide binding oligomerization domain (NOD)-like receptors (NLRs), retinoic acid inducible gene I-like receptors (RLRs), C-type lectin receptors (CLRs), and peptidoglycan recognition proteins (PGRPs) [2].

PGRPs were first identified in the silkworm *Bombyx mori* and named according to their high-affinity binding to PGN [3]. PGRPs were subsequently found to be conserved in the whole animal kingdom including insects [4], deuterostomes [5], fish [6] and mammals [7]. However, the copy numbers of PGRP genes vary in species, e.g. 13 PGRP genes in *Drosophila melanogaster* [4], 3 in zebrafish *Danio rerio* [6] and 4 in mammals [7]. Based on the length of peptide sequences, PGRPs are classified into three groups: short (S) PGRPs, intermediate (I) PGRPs and long (L) PGRPs, among which the short and long PGRPs are identified in all the invertebrates and vertebrates [4-7], while the intermediate PGRPs only exist in mammals [7]. All PGRPs contain at least one C-terminal PGRP domain of 165 amino acids. Structurally, PGRPs contain multi β-sheets and α-helices, which form an L-shaped groove involved in PGN binding [8].

Invertebrate PGRPs are crucial PRRs in antimicrobial innate immunity [9]. *Drosophila* PGRP-SA, PGRP-SD and PGRP-SC1 recognize PGN and subsequently activate the Toll pathway [10-12]. In contrast, *Drosophila* PGRP-LC activates the death-domain-containing Imd protein, inducing antimicrobial peptides to eliminate bacteria [13]. Silkworm PGRP-S is shown to bind bacteria PGN to activate the prophenol-oxidase cascade, generating melanin and reactive oxygen species to combat infections [3]. Furthermore, *Drosophila* PGRP-SC1 and PGRP-LB have N-acetylmuramoyl-L-alanine amidase activity of degrading bacterial PGNs [14, 15]. Studies have shown that teleost PGRPs have comparable functions of invertebrate orthologs. Zebrafish recombinant PGRPs are potent bactericidal agents against Gram-positive and Gram-negative bacteria [16]. Unlike teleost counterparts, mammalian PGRPs that have amidase activity do not possess direct bactericidal activity, while those without amidase activity are bactericidal [17].

Amphibians are placed at a unique evolutionary point when the living environment is transited from aquatic to terrestrial habitats. Previously, we identified two types of PGRPs (short and long PGRPs) from *Xenopus tropicalis*, a model amphibian species, and showed that these two PGRPs were up-regulated following bacterial infection [18, 19]. The Chinese giant salamander (*Andrias davidianus*) is the largest extant urodela species and one of the primitive amphibians. Information regarding salamander PGRPs is still scarce. In this study, a short PGRP (AdPGRP1) was cloned from salamander, and its expression patterns, amidase activity, PGNs binding ability, antimicrobial activity, and involvement in regulation of NF-κB pathway were studied. This work provides a basis for further analysis of the functions of amphibian PGRPs and the evolutionary history of animal PGRPs.

## RESULTS

### Sequence features of AdPGRP-S1

The cloned AdPGRP-S1 cDNA sequence (GenBank accession number: MF563613) was 985 bp in length, containing 131 bp of 5’-untranslated region (UTR), 555 bp of open reading frame (ORF) and 299 bp of 3’-UTR. AdPGRP-S1 cDNA sequence contained two stop codons in the 5’-UTR upstream of the start codon (ATG) and two in-frame stop condons in the 3’-UTR downstream of the stop condon, indicating that a complete ORF of AdPGRP-S1 had been obtained. The ORF of AdPGRP-S1 encoded 184 amino acids with a signal peptide of 17 aa predicted by the SignalP software. No N-glycosylation site and transmembrane domain were found (Supplementary Figure 1).

**Figure 1:**
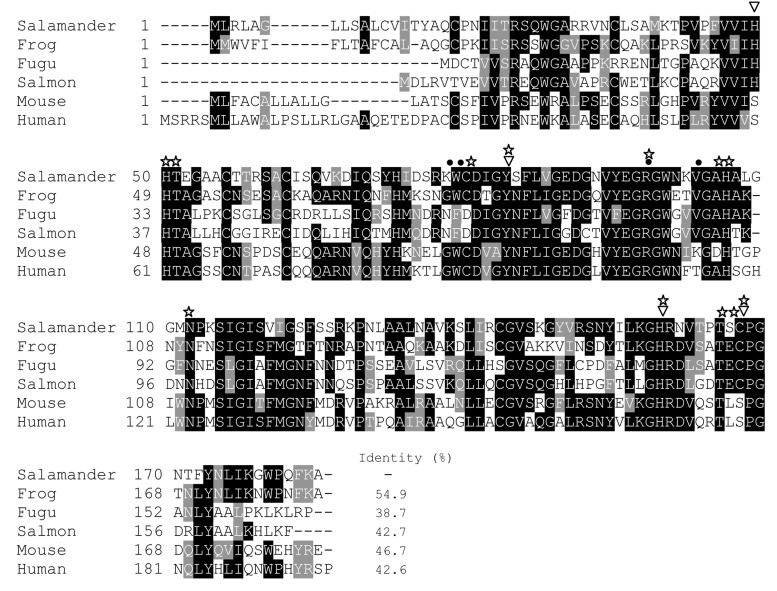
Multiple alignment of the amino acid sequences of vertebrates’ short PGRPs The sequence alignment was performed using the Clustal O software and edited with the BoxShade software. Sequence identity was analyzed using MegAlign in the DNAStar software package. Catalytic residues responsible for amidase activity and specific PGN recognition activity are marked by inverted white triangles and dots, respectively. The possible substrate binding sites were marked by white asterisks.

AdPGRP-S1 possesses a PGRP domain (residues 31 to 169). Notably, several conserved residues important for the PGRP functions were identified in the PGRP domain, including four catalytic residues responsible for amidase activity (H49, Y84, H159 and C167), four residues involved in specific PGN recognition activity (K78, W79, R98 and V103), and ten possible substrate binding sites (H50, T51, C80, Y84, R98, A105, H106, N112, H159, T165, S166 and C167) (Figure 1). AdPGRP-S1 shared 38.7%-54.9% sequence identities with vertebrates’ short PGRPs, with the highest identity (54.9%) with frog PGRP-S.

**Figure 2:**
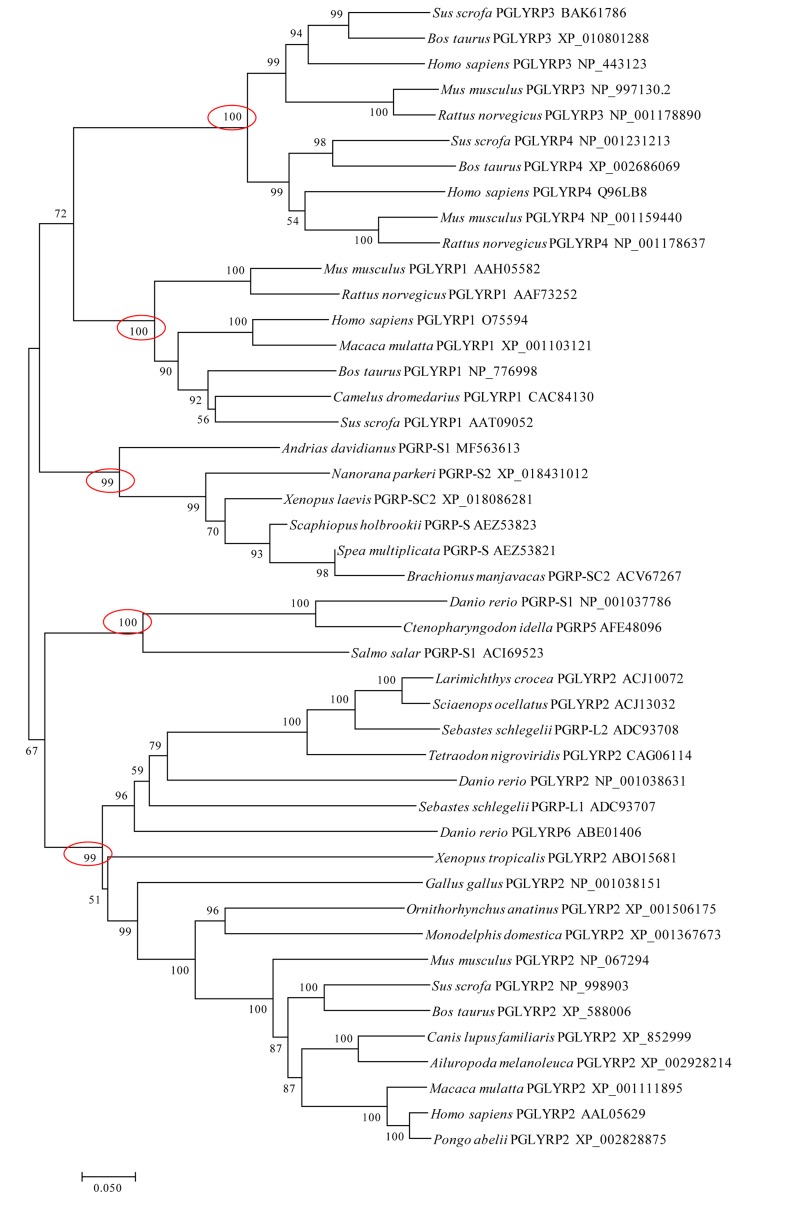
Evolutionary relationships of vertebrates’ PGRPs The evolutionary history was inferred using the Neighbor-Joining method in MEGA7 software. The percentages of replicate trees in which the associated taxa clustered together in the bootstrap test (10,000 replicates) were shown next to the branches. The evolutionary distances were computed using the JTT matrix-based method and were shown in the units of the number of amino acid substitutions per site.

To further understand the evolution of vertebrates’ PGRPs, an un-rooted phylogenetic tree was constructed using the MEGA 7.0 software based on the multiple sequences alignment (Figure 2). The phylogenetic tree was divided into five main clades, supported by the high bootstrap values (≥ 99%). They included the mammalian intermediate PGRPs (PGLYRP3/4), mammalian short PGRPs (PGLYRP1), the amphibian short PGRPs, which contained AdPGRP-S1, the teleost short PGRPs, and the vertebrates’ long PGRPs (Figure 2).

### Tissue distribution of AdPGRP-S1

**Figure 3:**
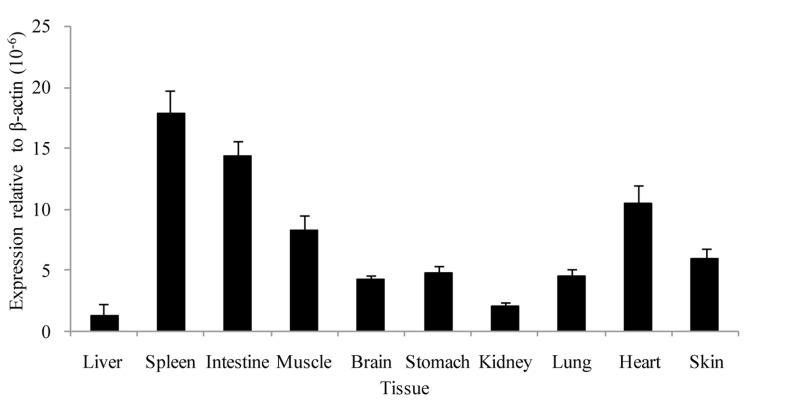
Tissue distribution of AdPGRP-S1 in selected tissues of normal salamanders Expression of AdPGRP-S1 was analyzed by real-time qPCR and normalized to β-actin in each tissue of 4 animals.

Real-time qPCR analysis demonstrated that AdPGRP-S1 was ubiquitously expressed in tissues, with the highest expression seen in spleen and intestine, and lowest in liver and kidney. Moderate expression of AdPGRP1 was also detected in muscle, heart, skin and lung (Figure 3).

### Analysis of the AdPGRP-S1 fusion protein expressed in HEK-293T cells

**Figure 4:**
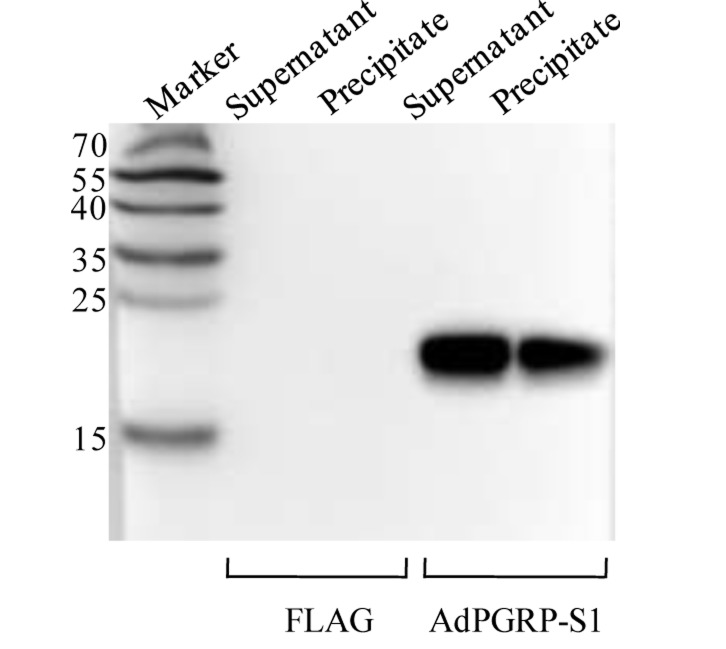
The extracellular and intracellular expression of AdPGRP-S1 in HEK-293T cell medium and lysates by western-blotting The numbers on the left panel indicate the size (kDa) of the molecular markers.

The AdPGRP-S1 fusion protein expressed in the HEK-293T cells was analyzed by Western blotting. A specific band of 15-25 kDa was detected in the supernatant and cell lysate of cells transfected with AdPGRP-S1 plasmid but not empty p3xFLAG vector, which was in line with the predicted molecular weight of AdPGRP-S1 (18 kDa). The results indicate that the AdPGRP1 fusion protein could be expressed as preprotein in intracellular region and could be secreted (Figure 4).

### PGNs binding ability of AdPGRP-S1

**Figure 5:**
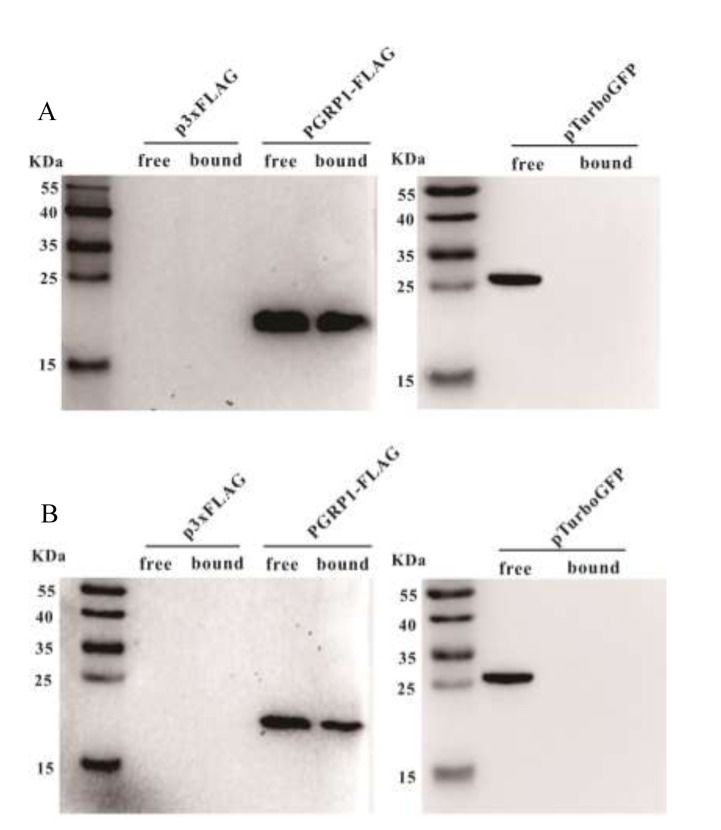
AdPGRP-S1 binding insoluble Lys-PGN from *Staphylococcus aureus* and Dap PGN from *Bacillus subtilis* Lysates of HKE-293T cells transiently transfected with p2xFLAG-CMV-14 or pPGRP-S1-FLAG plasmids were incubated with 40 μg Lys PGN from *S. aureus* or Dap PGN from *B. subtilis*, respectively. Proteins eluted from the pellets were separated by SDS-PAGE and detected using Western blotting with anti-FLAG antibody.

Four residues involved in PGN binding were identified in AdPGRP-S1 (Figure 1), demonstrating that AdPGRP-S1 might have PGN binding ability. To test this, p3xFLAG-CMV-14 or pPGRP-S1-FLAG plasmids were transiently transfected into HEK-293T cells and recombinant proteins were extracted and incubated with insoluble Lys-type PGN and DAP-type PGN, respectively. Fig. 4 showed that AdPGRP-S1 could bind both Lys-type PGN and DAP-type PGN. No band was detected in cells transfected with empty p3xFLAG vector (Figure 5).

### Amidase activity of AdPGRP-S1

**Figure 6:**
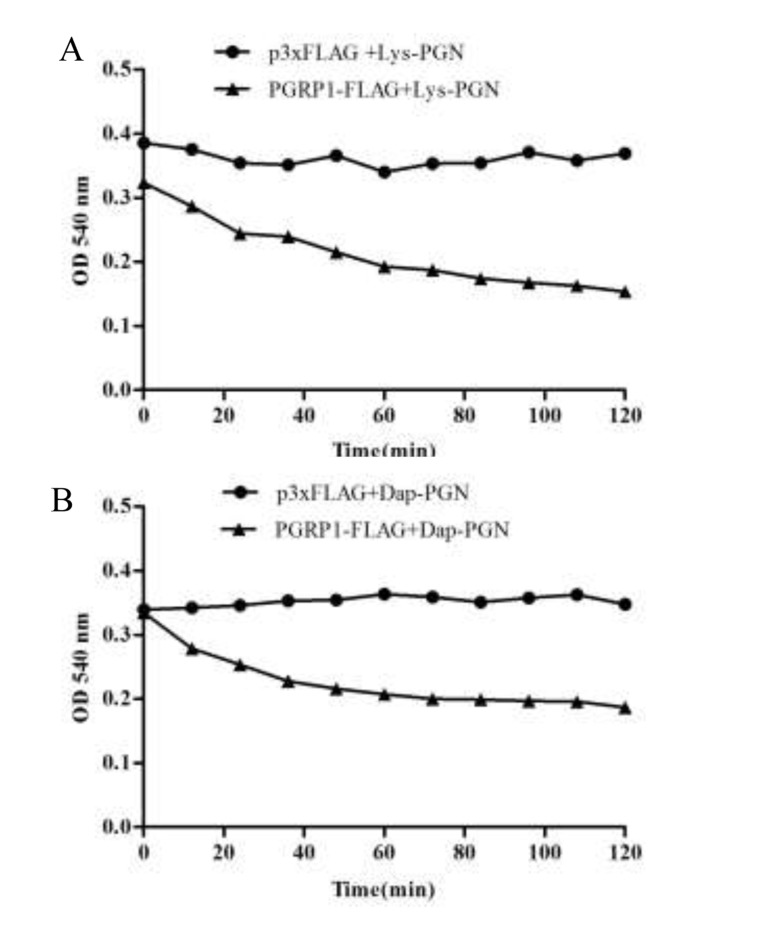
Degrading of AdPGRP-S1 on Lys PGN and Dap PGN Lysates of HKE-293T cells transiently transfected with p3xFLAG-CMV-14 and pPGRP-S1-FLAG plasmids were respectively incubated with Lys PGN and Dap PGN and the optical density (OD) at 540 nm was recorded every 5 minutes until 120 min post incubation.

Next, the amidase acitivity of AdPGRP-S1 on Lys-PGN and DAP-PGN was examined by assessing the optical clearance of solution at 540 nm [20]. In the presence of 10 μM Zn^2+^, apparent drop tendency was observed within 120 min when the lysates of pPGRP-S1-FLAG-transfected HEK-293T cells were incubated with Lys-PGN and DAP-PGN. No drop tendency was detected for the cell lystate of the control cells transfected with p3xFLAG plasmid (Figure 6).

### Antibacterial activity of AdPGRP-S1

**Figure 7:**
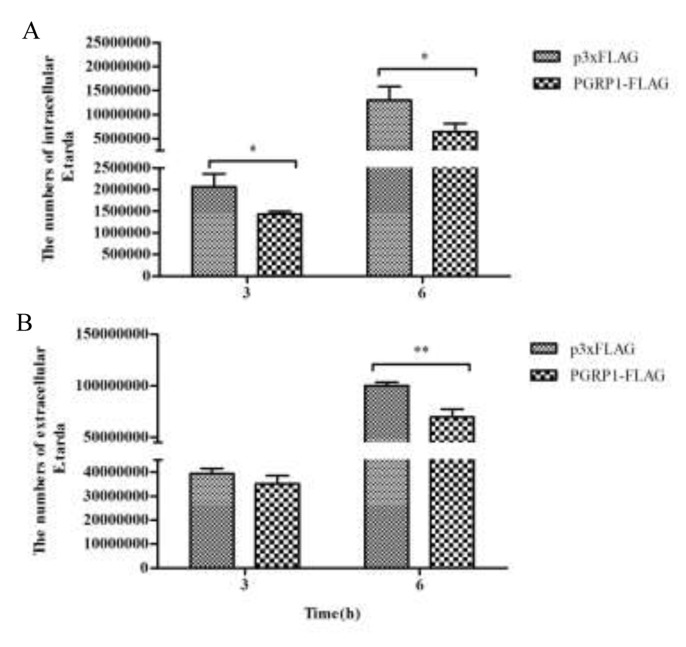
Inhibition of intracellular (A) and extracellular (B) *E.tarda* by AdPGRP-S1 HKE-293T cells transiently transfected with p3xFLAG-CMV-14 or pPGRP-S1-FLAG plasmids were infected with *E.tarda*. The numbers of bacteria were calculated at 3 h and 6 h post bacterial infection or post incubation with gentamicin. *P<0.05. **p < 0.01.

The AdPGRP-S1 fusion protein was shown to be expressed in intracellular region and also secreted. These intracellular and extracellular isoforms of AdPGRP-S1 were investigated for their antibactericidal effects. In cells transfected with AdPGRP-S1, it was observed that at 3 h and 6 h post *E. tarda* infection the numbers of intracellular bacteria were significantly lower than that of control cells transfected with p3xFLAG plasmids (Figure 7A). Similarly, the numbers of extracellular bacteria were significantly decreased in HEK-293T cells transfected with AdPGRP-S1 at 6 h post *E. tarda* infection (Figure 7B).

### Activation of NF-κB by AdPGRP-S1

**Figure 8:**
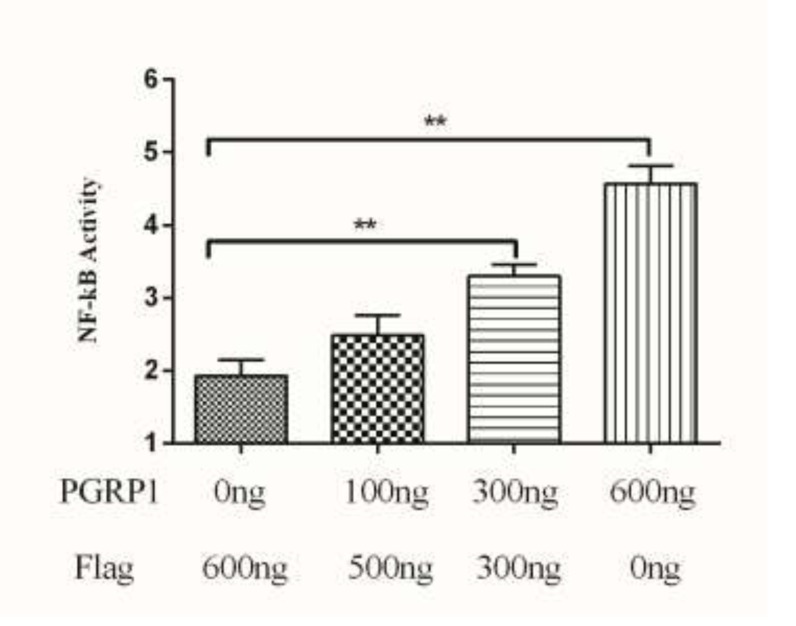
Effects of AdPGRP-S1 overexpression on the activity of the NF-кB reporter gene The HEK-293T cells were transiently co-transfected with pRL-TK, NF-κB reporter vector, and pPGRP-S1-FLAG expression vector. The p3xFLAG-CMV-14 vector was used as a control. **p < 0.01.

The NF-κB signaling pathway is important in regulating innate and adaptive immune responses [21]. The PGRPs of teleost and mammals mediate NF-κB pathway. We hypothesized that AdPGRP-S1 might also be involved in the NF-κB pathway and tested the effect of AdPGRP1-S1 on the activation of NF-κB in HEK-293T cells using a luciferase reporter gene assay. The results confirmed that the NF-κB luciferase reporter was activated by pPGRP-S1-FLAG in a dose-dependent manner, with a maximum increase of 4.5-fold relative to transfection of HEK-293T cells with p3xFLAG-CMV-14 (control) alone (*P* < 0.01) (Figure 8). These results indicated that AdPGRP-S1 could trigger the activation of the NF-κB signaling pathway in HEK-293T cells.

## DISCUSSION

Chinese giant salamander (*A.davidianus*), the largest extant urodela amphibian species, has substantial scientific and economic values. However, little is known about its immune defence. In the present study, we cloned and functionally analyzed a short PGRP (AdPGRP-S1) in this species. To our best knowledge, this was the first report on the functions of PGRP in salamander. The cloned AdPGRP-S1 was 184 aa in length, which had similar sequence length with invertebrates’ and vertebrates’ short PGRPs [4, 6, 7]. Multiple sequence alignment showed that AdPGRP-S1 shared high sequence identities (38.7%-54.9%) with other vertebrates’ short PGRPs. Similar to other vertebrates’ PGRPs, AdPGRP-S1 also contained one typical PGRP domain at its C-terminal region. The length of PGRP domain of AdPGRP-S1 (138 aa) was comparable to that of other vertebrates’ PGRPs (∼ 165 aa) [9]. In the PGRP domain of AdPGRP-S1, several residues involved in functions of PGRPs were identified, including four catalytic residues involved in amidase activity, four residues involved in specific PGN recognition activity and ten possible substrate binding sites. These structural features revealed that AdPGRP-S1 might have functions of bacterial recognition and effector to eliminate pathogens.

The expression of AdPGRP-S1 in tissues of normal salamander was analyzed by quantitative real-time PCR, providing clues to understand its functions. Results showed that AdPGRP-S1 was ubiquitously expressed in all the tissues analyzed, in agreement with previous studies [6, 7, 18, 19]. Similar to fish species, salamanders also live in aquatic environment. Constitutive expression of AdPGRP-S1 in tissues might be favorable to combat the aquatic pathogens. It was interesting that AdPGRP-S1 was highly expressed in immune related tissues. We also found that AdPGRP-S1 was highly expressed in salamander intestine and skin which are important tissues of mucosal immunity [21, 22]. In addition, AdPGRP-S1 was expressed in non-immune tissues, e.g. muscle, heart. Similar results were also found for PGRPs of invertebrates [23] and teleost [24], suggesting that PGRPs might also play roles in processes other than immune defense against bacterial pathogens.

Genes of PGRPs could encode secreted proteins, membrane proteins and intracellular proteins. All of *Drosophila* short PGRPs were secreted proteins, among which PGRP-SA and PGRP-SD acted as pattern recognition receptors, PGRP-SB and PGRP-SC had amidase activity to hydrolyze PGN [25]. The four mammalian PGRPs were also secreted proteins, which were differently expressed and involved in immune responses in different tissues [7]. Zebrafish PGLYRP2, PGLYRP5 and PGLYRP6, grass carp (*Ctenopharyngodon idella*) PGLYRP6 were also secreted proteins [16, 26]. Similar to human PGLYRP1 [7], AdPGRP-S1 also possessed a signal peptide at its N-terminus and was detected as a secretory protein. Signal peptide is essential for the classic protein secretion pathway where proteins were transported through endoplasmic reticulum (ER) and Golgi to the extracellular membrane [27]. We speculate that AdPGRP-S1 might be secreted through the classic protein secretion pathway.

PGRPs were firstly named according to the ability to bind PGN [3]. However, different types of PGRPs showed selective binding ability towards Lys-PGN and Dap-PGN. It had been found that the three important amino acids located in the PGN-binding cleft of PGRPs were essential for classification of the PGN types bound by PGRPs [28]. For example, human PGLYRP1 containing Gly89, Trp90 and Arg109 could bind Dap-type PGN, PGLYRP3 with Asn236, Phe237 and Val256 could bind Lys-type PGN [9], grass carp PGLYRP5 with Gly73, Phe74 and Arg93 could bind both types of PGNs [29]. In the present study, we found that AdPGRP-S1 contained Lys78, Trp79 and Arg98 and was able to bind both types of PGNs. Also, the different combinations of these three amino acids in the PGN-binding cleft of PGRPs were found in other species. The relationship between the amino acids and PGN binding specificity requires further investigations.

Some PGRPs had amidase activity which converted pro-inflammatory PGNs into non-immunogenic fragments. These included *Drosophila* PGRP-SB1 and PGRP-LB, and mammalian PGLYRP2 [15, 30, 31]. All the PGRPs possessing amidase activity contained four conserved Zn^2+^ binding sites, involving several key amino acid residues, e.g. His98, Tyr132, His206 and Cys214 in zebrafish PGLYRP5 [16]. Zn^2+^ acts as electrophilic catalyst during the hydrolytic process of PGN, promoting the hydrolysis of bond between the lactyl group of the N-acetylmuramic acid and the L-alanine of peptide [15, 30, 31]. The four Zn^2+^ binding sites played essential roles in the catalytic activity of PGRPs. Mutant forms of human PGLYRP2 (C530S), *Drosophila* PGRP-SC1b (C168A and C168S) were shown to lack amidase activity [20]. In this study, we found that AdPGRP-S1 also contained four conserved Zn^2+^ binding sites and was capable of degrading PGNs, suggesting that it possesses both PGN binding activity and amidase activity, similar to that of teleost PGRPs [16].

We demonstrated in the present study that AdPGRP-S1 possesses antibacterial activities, inhibiting proliferation of a pathogenic Gram-negative bacterium. This was in agreement with studies in teleosts and mammals [16, 32]. Notably, the antibacterial mechanisms of teleost and mammalian PGRPs are different. Mammalian bactericidal PGRPs do not have amidase activity, indicating the bactericidal activity of mammalian PGRPs is independent of amidase activity [32, 33]. Unlike mammalian PGRPs, fish PGRPs possess both amidase and bacterial activity [16]. Whether insect PGRPs are bactericidal is less clear, except for *Drosophila* PGRP-SB1 that possesses both amidase and bacterial activities [34]. These observations suggest that teleost and insect PGRPs might be amidase-dependent, involving in peptidoglycan hydrolysis or permeabilization of the cytoplasmic membrane [16]. The AdPGRP-S1 also possesses both amidase and bactericidal activity, and could function within the cells and extracellularly.

Our study demonstrated that AdPGRP-S1 could trigger the activation of the NF-κB signaling pathway. A dose-dependent induction of NF-κB promoter in transfected cells was seen. Similar results were also found in fish PGRPs. For example, rainbow trout PGRP-L1 could inhibit the activation of the NOD-induced NF-κB pathway via downregulation of TAK1 and IκBα phosphorylation [35]. Zebrafish PGRP-SC could regulate expresssion of several immune related genes, including TLR2, TLR3, interleukin (IL)-17 and NF-κB [36].

In summary, we cloned a short PGRP from the Chinese giant salamander and analyzed its functions for the first time. AdPGRP-S1 was constitutively expressed in tissues of normal salamander. Functional analysis revealed that AdPGRP-S1 had similar activities as teleost PGRPs, including PGN binding activity, antibacerial activity, amidase activity and signaling regulation. The results suggest that the functional divergence of amidase activity and PGN binding activity of verterbrates’ PGRP might have taken place after the emergence of tetrapods.

## MATERIALS AND METHODS

### Ethics statement

This study was approved by the Ethics Committee of Animal Experiments (Institute of Hydrobiology; Permit Number: Y213531301). All surgery was performed under anesthesia using 500 mg/L gethyl-3-aminobenzoate methanesulfonate (MS-222, Sigma, USA).

### Cloning cDNA of AdPGRP-S1

Normal Chinese giant salamanders (average body weight 200 g) were obtained from a farm in Hubei province, P. R. China. Total RNA was extracted from the liver of normal salamanders using Trizol reagent (Invitrogen, USA) according to manufacturer’s instructions. cDNA was then synthesized using First Strand cDNA Synthesis Kit (Thermo Scientific, USA). Specific primers (AdPGRP1F and AdPGRP1R) were designed according to the partial sequence obtained from the salamander transcriptome [37]. Then, 3’-RACE and 5’-RACE were performed using gene specific primers and adaptor primers (UPM) to obtain the full cDNA length of AdPGRP-S1.

### Cell line, bacteria and reagents

The human embryonic kidney cell line (HEK-293T) was maintained in MEM medium (Gibco, USA) supplemented with 10% fetal bovine serum (FBS) (Sigma, USA) at 37°C. *Edwardsiella tarda* (strain PPD130/91) was used for antibacterial activity assay. Lys-type PGN (catalog no. 77140) from *Staphylococcus aureus* and Dap-type PGN (catalog no. 69554) from *Bacillus subtilis* were purchased from Sigma-Aldrich (USA).

### Sequence and phylogenetic analyses

Alignment of multiple sequences was performed using the Clustal Omega software (http://www.ebi.ac.uk/tools/msa/clustalo) and edited with the BoxShade software (http://www.ch.embnet.org/software/BOX_form.html). Protein sequence identity was calculated by the MatGat 2.02 software [38]. The signal peptide and transmembrane domain were predicted by the SignalP 4.1 server [39] and TMHMM server 2.0 [40], respectively. Various physical and chemical parameters including molecular weight, theoretical isoelectric point, amino acid composition, atomic composition, extinction coefficient, estimated half-life, instability index, aliphatic index and grand average of hydropathicity (GRAVY) of proteins were analyzed using ProtParam tool [41]. The protein domains were searched in the Pfam database [42]. A phylogenetic tree was constructed using the Neighbor-Joining (N-J) method in MEGA 7.0 software, with bootstrapping set as 10,000 repetitions to assess the reliability of branch topology.

### Tissue distribution of AdPGRP-S1

To investigate the tissue distribution of AdPGRP-S1, ten tissues including liver, spleen, intestine, muscle, brain, stomach, kidney, lung, heart and skin were collected from four normal salamanders. Total RNA was extracted from these tissues using Tirzol reagent (Invitrogen, USA), and reverse transcribed into first strand cDNA using PrimeScript^®^ RT reagent Kit with gDNA Erase (Takara, Japan). Real-time qPCR was performed using SYBR Green fluorescent dye (Invitrogen, USA) on the CFX96 Touch™ Real-Time PCR Detection System (Bio-Rad, USA) and analyzed as described previously [43, 44]. The expression level of AdPGRP-S1 was normalized to that of β-actin. Primers used for real-time quantitative PCR are listed in Table 1.

**Table 1:** Primers used in this study.

Primer	Sequence (5' to 3')	Usage
AdPGRP1F	TGCACCACCAGATCCGCCTG	Gene clone
AdPGRP1R	GGGCAACTGGTGGGCGTCA	Gene clone
AdPGRP1-5out	CCCAACTTTGTTCCAGCCGCG	5' RACE PCR
AdPGRP1-5in	GGAAGCTGTAGCCAATATCACAC	5' RACE PCR
AdPGRP1-3out	CCTTGGCGGCATGAACCCCA	3' RACE PCR
AdPGRP1-3in	CGGTCCAACTACATCCTGAAGG	3' RACE PCR
Long-UPM	CTAATACGACTCACTATAGGGCAA- GCAGTGGTATCAACGCAGAGT	RACE PCR
Short-NUP	CTAATACGACTCACTATAGGGC	RACE PCR
AdPGRP1-F	CAAACTTGGCCGCCCTGAACG	Realtime PCR
AdPGRP1-R	CCTTGAACTGCGGCCACCCT	Realtime PCR
Adactin-F	CCACTGCTGCCTCCTCTT	Realtime PCR
Adactin-R	GCAATGCCTGGGTACATG	Realtime PCR
AdPGRP1F1	CCCAAGCTTATGCTGCGTCTGGCAGGGC	Plasmid construction
AdPGRP1R1	CGGGATCCGGCCTTGAACTGCGGCCAC	Plasmid construction

### Construction of expression vectors for the production of AdPGRP-S1 fusion protein

The open reading frame (ORF) of AdPGRP-S1 was amplified with primers (AdPGRP1F1 and AdPGRP1R1) and inserted into the *Hind III* and *BamH I* sites of p3xFLAG-CMV-14 (Sigma, USA) to generate pPGRP-S1-FLAG expression plasmid.

### Expression analysis of the AdPGRP-S1 fusion protein

The HEK-293T cells were used to study the function of AdPGRP-S1. The p3xFLAG-CMV-14 or pPGRP-S1-FLAG plasmids were transiently transfected into 2 × 10^6^ HEK-293T cells using LipofectAMINE 2000 transfection reagent (Invitrogen, USA) following the manufacture’s construction. After 48 h, the cell media and cell pellet were collected and the expression of AdPGRP-S1 in cell media and cell pellet were detected with anti-FLAG antibody by Western blotting.

### PGNs binding assay

Three micrograms of p3xFLAG or pPGRP-S1-FLAG plasmids were transiently transfected into 2 × 10^6^ HEK-293T cells using LipofectAMINE 2000 transfection reagent (Invitrogen, USA) following the manufacture’s construction. After 48 h, protein was extracted using RIPA buffer (plus protease inhibitor cocktails) (Thermo Scientific, USA) and frozen at -80°C. Forty micrograms of insoluble Lys-type PGN and DAP-type PGN were incubated with 50 μg extracted proteins for 4 h at 4°C in a rocking incubator. Then, the bound and unbound proteins were separated by centrifugation at 13,000 rpm for 15 min and washed 4 times with TBS buffer (50 mM TrisHCl, 50 mM NaCl, 10 μM ZnCl_2_, pH 7.5). The bound proteins were recovered from PGN by boiling in 2 × SDS-PAGE loading buffer. Then, the proteins were loaded onto the SDS-PAGE gels for gel electrophoresis and detected with anti-FLAG antibody by Western blotting.

### Amidase activity analysis

The p3xFLAG or pPGRP-S1-FLAG plasmids were transfected into HEK-293T and proteins were extracted as mentioned above. Forty micrograms of insoluble Lys-type PGN and DAP-type PGN were incubated with 50 μg extracted proteins in Tris-ZnCl_2_ buffer (20 mM Tris HCl, 150 mM NaCl, 10 μM ZnCl_2_, pH 7.2). The PGNs incubated with p3xFLAG-CMV-14 in Tris-ZnCl_2_ buffer were set as control groups. The optical density (OD) at 540 nm was recorded every 5 minutes until 120 min post incubation.

### Antibacterial assay

#### Intracellular antibacterial assay

The 3 × 10^5^ HEK-293T cells were transiently transfected with 0.5 μg p3xFLAG or pPGRP-S1-FLAG and cultured at 37 °C for 48 h. The cells were infected with *E. tarda* at the multiplicity of infection (MOI) of 10 (2 × 10^7^ cfu/ml) for 1 h at 25 °C. After the cells were washed with DMEM medium for three times, the 10% FBS DMEM medium containing 16 μg/ml gentamicin was added to each well to kill extracellular bacteria. The cells were collected at 3 h and 6 h post incubation with gentamicin. The cells were washed with DMEM medium for four times and then lysed in 500 μl PBS containing 1% Triton X-100 for 20 min. The numbers of bacteria were calculated by plate colony-counting methods.

#### Extracellular antibacterial assay

The 3 × 10^5^ HEK-293T cells were transiently transfected with 0.5 μg p3xFLAG or pPGRP-S1-FLAG and cultured at 37 °C for 48 h, and the cells were then infected with *E. tarda* at the multiplicity of infection (MOI) of 10 (2 × 10^7^ cfu/ml) for 1 h at 25 °C. At 3 h and 6 h post infection, the medium were collected and the numbers of bacteria were calculated by plate colony-counting methods.

No additional antibiotics were used during the intracellular and extracellular antibacterial experiments. All the intracellular and extracellular antibacterial experiments were performed in triplicate and the data were expressed as mean ± SD, and analyzed using Student’s t-test with *P* < 0.05 considered statistically significant.

### Transient transfection and luciferase reporter assays

Activation of the NF-κB pathway was measured using the luciferase reporter assay as described previously [26]. The HEK-293T cells were transfected with 100 ng NF-κB luciferase plasmid, 10 ng pRL-TK vector and different concentrations of pPGRP-S1-FLAG plasmids (100, 300 and 600 ng). The p3xFLAG vector was used as control. At 36 h post transfection, the cells were lysed and the luciferase activity was measured. The experiments were performed in triplicate and the data were expressed as mean ± SD, and statistical difference was determined using Student’s t-test at *P* < 0.05.

## SUPPLEMENTARY MATERIALS FIGURE


