# Supplementary material for: Functional characterization of a short peptidoglycan recognition protein from Chinese giant salamander (Andrias davidianus)
Source: Oncotarget. 2017 Oct 3;8(59):99323–35. doi: 10.18632/oncotarget.21470 (PMC5725095; doi:10.18632/oncotarget.21470)
Supplement: Supplementary file 1 [file oncotarget-08-99323-s001.pdf]

Functional characterization of a short peptidoglycan recognition protein from Chinese giant salamander (*Andrias davidianus)*

SUPPLEMENTARY MATERIALS

A

```
  1 cctctattacaggaaagaccagaatctagtgctcctcctcagccacataccaggacccgt
 61 gggacgaaggctagaccaggtcaccggttgtgctgttgtcttctcggggatttactaga
121 acgttgccgggATGCTGCGTCTGGCAGGGCTGCTCTCTGCTCTGTGTGTCATCACATATG
  1             M  L  R  L  A  G  L  L  S  A  L  C  V  I  T  Y  A
181 CACAGTGCCCCAACATTATCACCCGCTCGCAGTGGGGAGCCCGCAGGGTCAACTGCCTCA
 18  Q  C  P  N  I  I  T  R  S  Q  W  G  A  R  R  V  N  C  L  S
241 GTGCCATGAAGACGCCAGTACCATTTGTCGTCATCCATCACACAGAAGGAGCAGCCTGCA
 38   A  M  K  T  P  V  P  F  V  V  I  H  H  T  E  G  A  A  C  T
301 CCACCAGATCCGCCTGCATCAGCCAGGTCAAGGACATCCAGAGCTACCACATTGACAGCA
 58   T  R  S  A  C  I  S  Q  V  K  D  I  Q  S  Y  H  I  D  S  R
361 GGAAATGGTGTGATATTGGCTACAGCTTCCTAGTTGGCGAGGATGGCAATGTTTACGAAG
 78   K  W  C  D  I  G  Y  S  F  L  V  G  E  D  G  N  V  Y  E  G
421 GACGCGGCTGGAACAAAGTTGGGGCCCACGCCCTTGGCGGCATGAACCCCAAGTCAATTG
 98   R  G  W  N  K  V  G  A  H  A  L  G  G  M  N  P  K  S  I  G
481 GCATCAGCGTCATCGGCTCCTTCTCAAGCAGAAAACCAAACTTGGCCGCCCTGAACGCAG
118   I  S  V  I  G  S  F  S  S  R  K  P  N  L  A  A  L  N  A  V
541 TGAAGAGTCTCATCCGCTGTGGGGTCTCCAAGGGCTACGTTCGGTCCAACTACATCCTGA
138   K  S  L  I  R  C  G  V  S  K  G  Y  V  R  S  N  Y  I  L  K
601 AGGGTCACCGTAATGTGACGCCCACCAGTTGCCCGGGGAACACTTTCTACAACCTCATCA
158   G  H  R  N  V  T  P  T  S  C  P  G  N  T  F  Y  N  L  I  K
661 AAGGGTGGCCGCAGTTCAAGGCCTAAgtgttggctccaggccagggaaccggcggagaag
178   G  W  P  Q  F  K  A  *
721 cggattgaaaccagcttaaaatgccaaagccttgcaattctcatacatgttcaagtccac
781 cttctttaagctcacgtttgtgccatgttatccttgattatccaccagcttcccccccc
841 ccaccatgctaccccgtaaggtacccttgttctatcatgtctcatgggtcacctctttca
901 ccaaattaaacataagctctacggggcagaagcggtgcctgtctgatggcattattgtaa
961 gctccgcagggcagtgagcggcggg
```

B

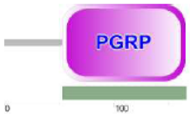

Supplementary Figure 1: Nucleotide and deduced amino acid sequence (**A**) and domain organization (**B**) of adPGRP-S1**.** the start codon (ATG) and stop codon (TGA) were boxed. The in-frame stop codons updtream of the open reading frame were underlined and marked in gray color. The predicted signal peptide was double underlined. The amino acid sequences of PGRP domain (residue 31 to 169) was marked in grey.
